# 

**Published:** 1890-09

**Authors:** 


*Cases for binding, price 7d. each, may be had of the Publisher,*
J. W. ARROWSMITH, *Quay Street, Bristol.*

### *PUBLICATIONS RECEIVED.*

From Alexander and Shepheard, London:
*The Care of the Skin.* By F. Augustus Cox, M.B. 1890.

From Baillière, Tindall & Cox, London:
*Dental Surgery.* By Henry Sewill. Third Edition. 1890.

From Ev. E. Carreras, St. Louis:
*The Alienist and Neurologist.* July, 1890.

From Alfred Hölder, Vienna:
*Zur Ichthyolbehandlung von Frauenkrankheiten.* Von Dr. Reitmann und Dr. Schönauer. 1890. Reprint.

From H. K. Lewis, London:
*The Shadow-Test.* By W. M. Beaumont. 1890.
*Diseases of the Nose.* By James B. Ball, M.D. 1890.
*Masso-Therapeutics.* By William Murrell, M.D. Fifth Edition. 1890.
*The British Journal of Dermatology.* July—September, 1890.
*Hygiene and Public Health.* By Louis C. Parkes, M.D. Second Edition. 1890.
*Lymph-Stasis.* By Wayland C. Chaffey, M.D. 1890.
*Treatment of Diseases of the Nervous System.* By C. W. Suckling, M.D. 1890.
*Diseases of the Nose.* By W. Spencer Watson. Second Edition. 1890.

From Sampson Low, Marston, Searle & Rivington, Limited, London:
*Madeira and the Canary Islands.* By A. Samler Brown. Second Edition. 1890.

From Young J. Pentland, Edinburgh.
*Hypnotism.* By R. W. Felkin, M.D. 1890.

From Percival and Co., London:
*Influenza: An Historical Survey.* By E. Symes Thompson, M.D. 1890.

From W. H. Smith, Leamington:
*The Natural Mineral Waters and Spa of Leamington.* By R. Eardley-Wilmot, M.B. 1890.

From W. L. Smith, Detroit:
*Detroit Emergency Hospital Reports.* June, 1890.

From The Gazette Printing Co., Montreal:
*McGill University Annual Calendar.* Faculty of Medicine. Fifty-eighth Session. 1890.

From The General Publishing Company, Limited, London:
*The Magazine and Book Review.* No. 1. August 30th, 1890.

From John Thomlinson, Glasgow:
*Chloroform Syncope.* By Robert Kirk, M.D. 1890.

From 57 Park Street, London, W.:
*The Hamilton Association for Providing Trained Male Nurses. Fifth Annual Report.* 1890.

